# The Development and Validation of a Live Porcine Model for Endoscopic Endonasal Management of Internal Carotid Artery Injury With Fluorescence-Based Flow Assessment

**DOI:** 10.7759/cureus.99308

**Published:** 2025-12-15

**Authors:** Camila Dassi, Miguel Tepedino, Beatriz Sperini, Leonardo Balsalobre, Pedro Vianna, Aldo Stamm, Rogerio Pezato

**Affiliations:** 1 Otolaryngology, Universidade Federal de São Paulo (UNIFESP), São Paulo, BRA; 2 Otolaryngology, Universidade do Estado do Rio de Janeiro, Rio de Janeiro, BRA; 3 Otolaryngology, Hospital Edmundo Vasconcelos, São Paulo, BRA

**Keywords:** endoscopic endonasal surgery, fluorescein sodium, hemostasis, indocyanine green, internal carotid artery injury, muscle patch, porcine model, surgical education, vascular simulation

## Abstract

Background and objective

Internal carotid artery (ICA) injury during endoscopic endonasal skull base surgery is a rare but catastrophic complication. Simulation platforms are critical for training and for evaluating hemostatic strategies under realistic hemodynamic conditions. This study aimed to develop and validate a live porcine model for ICA injury that reproduces catastrophic hemorrhage, to compare vascular patency following repair with a crushed skeletal muscle patch versus an aneurysm clip, and to evaluate post-repair blood flow using endonasal endoscopic intraoperative fluorescence videoangiography.

Methods

Forty-five Large White pigs were studied between 2021 and 2024. Five animals were used in a pilot phase to assess the feasibility of the model and its hemodynamic reproducibility. In the experimental phase, 40 animals (80 carotid arteries) underwent standardized carotid injury, sequential hemostasis with crushed muscle and aneurysm clip, and perfusion assessment using indocyanine green (ICG) or fluorescein sodium (FNa). Outcomes included immediate hemostasis and distal vessel patency.

Results

All repairs achieved primary hemostasis. Patency was significantly higher after muscle patch (79/80, 98.8%) compared with aneurysm clip (64/80, 80.0%; McNemar p<0.001). Both ICG and FNa confirmed distal perfusion; despite unequal distribution of cases, FNa did not demonstrate statistical inferiority compared with the widely used ICG/near-infrared (ICG/NIR) system.

Conclusions

This porcine model reliably simulates catastrophic ICA injury and enables objective comparison of repair strategies. Crushed muscle preserved vessel patency at higher rates compared with aneurysm clip within this experimental model. Fluorescence angiography with ICG or fluorescein offers practical intraoperative flow verification, with fluorescein representing a cost-effective alternative. This model offers a consistent experimental environment that may support both translational research and surgical training.

## Introduction

Endoscopic endonasal approaches (EEA) have revolutionized access to skull base lesions by offering enhanced visualization, reduced morbidity, and faster recovery compared to traditional open techniques. However, internal carotid artery (ICA) injury remains the most dreaded intraoperative complication, with a substantial risk of catastrophic hemorrhage, stroke, pseudoaneurysm formation, and, in severe cases, death. Although infrequent, such events demand immediate, precise technical management, as the operative field is rapidly obscured and every second is critical for patient survival and neurological preservation [1,2]. The literature indicates that most surgeons performing expanded skull base procedures will ultimately face this scenario. However, the low incidence of injuries in routine practice limits opportunities for skill acquisition, preventing trainees and even experienced surgeons from developing proficiency under controlled conditions. This limitation highlights the need for experimental models that enable repetitive, standardized training in a safe environment where mistakes do not carry irreversible consequences [1,3].

Animal models have played a critical role in this context. The ovine model introduced by Valentine R and Wormald was groundbreaking, replicating high-flow arterial bleeding within a confined endonasal corridor and offering a realistic platform for evaluating hemostatic strategies [3,4]. Subsequent studies have validated the use of crushed skeletal muscle as an effective method for bleeding control and vascular patency preservation [2,5], as well as the use of aneurysm clips in selected cases. In parallel, intraoperative fluorescence imaging technologies using indocyanine green (ICG) and fluorescein sodium (FNa) have evolved, becoming valuable tools to evaluate vessel perfusion and integrity during and after repair [6,7].

In addition, the choice of swine as the experimental model has been guided by both practical and anatomical advantages. In Latin America, pigs are far more readily available for research than sheep, reflecting regional livestock production patterns and accessibility for experimental use [8]. From an anatomical standpoint, the porcine common carotid artery, with a reported diameter of approximately 4.0-5.8 mm, closely approximates the caliber of the human ICA in its parasellar and paraclival segments (around 5 mm). These features reinforce the translational relevance of the swine model for simulating endonasal vascular injury and testing hemostatic techniques [9,10].

Despite these advances, there has been no standardized live porcine model integrating these elements into a single protocol: controlled arterial injury creation, objective comparison of different hemostatic techniques, and fluorescence-based assessment of post-repair flow. The porcine model offers significant anatomical and physiological advantages, with arterial calibers and hemodynamic responses closely resembling those of humans, as well as greater availability in surgical training centers. This study aimed to describe in detail the development and validation of a live porcine model for simulating ICA injury during endoscopic endonasal surgery, evaluating two hemostatic techniques - crushed muscle patch and aneurysm clip - and documenting post-repair perfusion with intraoperative fluorescence imaging using ICG and FNa.

## Materials and methods

Ethical considerations

All animal procedures were conducted in strict accordance with Brazilian national regulations for the use of animals in research and followed the principles of the 3Rs (Replacement, Reduction, Refinement), the CONCEA guidelines, and the ARRIVE 2.0 reporting standards. Animals were housed under controlled temperature, humidity, and light-dark cycles, and all measures were taken to minimize stress before experimentation. Ethical approval was obtained from the São Paulo Federal University (UNIFESP) Institutional Animal Care and Use Committee, protocol CEUA 1280140223, and from the Institut de Recherche contre les Cancers de l’Appareil Digestif - Latin America Unit (IRCAD Rio de Janeiro) Animal Ethics Committee, protocols CEUA 5063151222, CEUA 9613090721, and CEUA 8530021221. To comply with the Reduction principles, both common carotid arteries were used in each animal, avoiding an unnecessary increase in sample size.

Animals and study design

A total of 45 Large White pigs were included in this prospective experimental study in a live porcine model. The study was divided into two phases. In the pilot phase, five animals were used to assess the feasibility and reproducibility of the porcine carotid injury model. Hemodynamic parameters recorded included weight, mean arterial pressure (MAP), and heart rate (HR) before and after injury, the time from carotid injury to cardiac arrest, infused fluid volume, cumulative blood loss, and MAP values at predefined time points. In the main experimental phase, 40 animals (80 carotid arteries) underwent a standardized protocol for carotid injury, sequential hemostasis, and intraoperative fluorescence evaluation.

Anesthesia and monitoring

Animals were fasted for 12 hours preoperatively. Sedation was induced with intramuscular midazolam (0.5 mg/kg) and ketamine (6.7 mg/kg). Orotracheal intubation was performed, and anesthesia was maintained with isoflurane (3.0 v% in oxygen) using mechanical ventilation. Intravenous midazolam (0.01 mcg/kg/min) was available if required. Norepinephrine (0.1 mcg/kg/min IV) was prepared for hemodynamic support when necessary. Monitoring included continuous electrocardiography, pulse oximetry, capnography, and invasive arterial pressure via femoral cannulation. At the end of the procedure, animals were euthanized under deep anesthesia with intravenous potassium chloride (2 mmol/kg), in accordance with AVMA guidelines.

Simulation model

**Figure 1:**
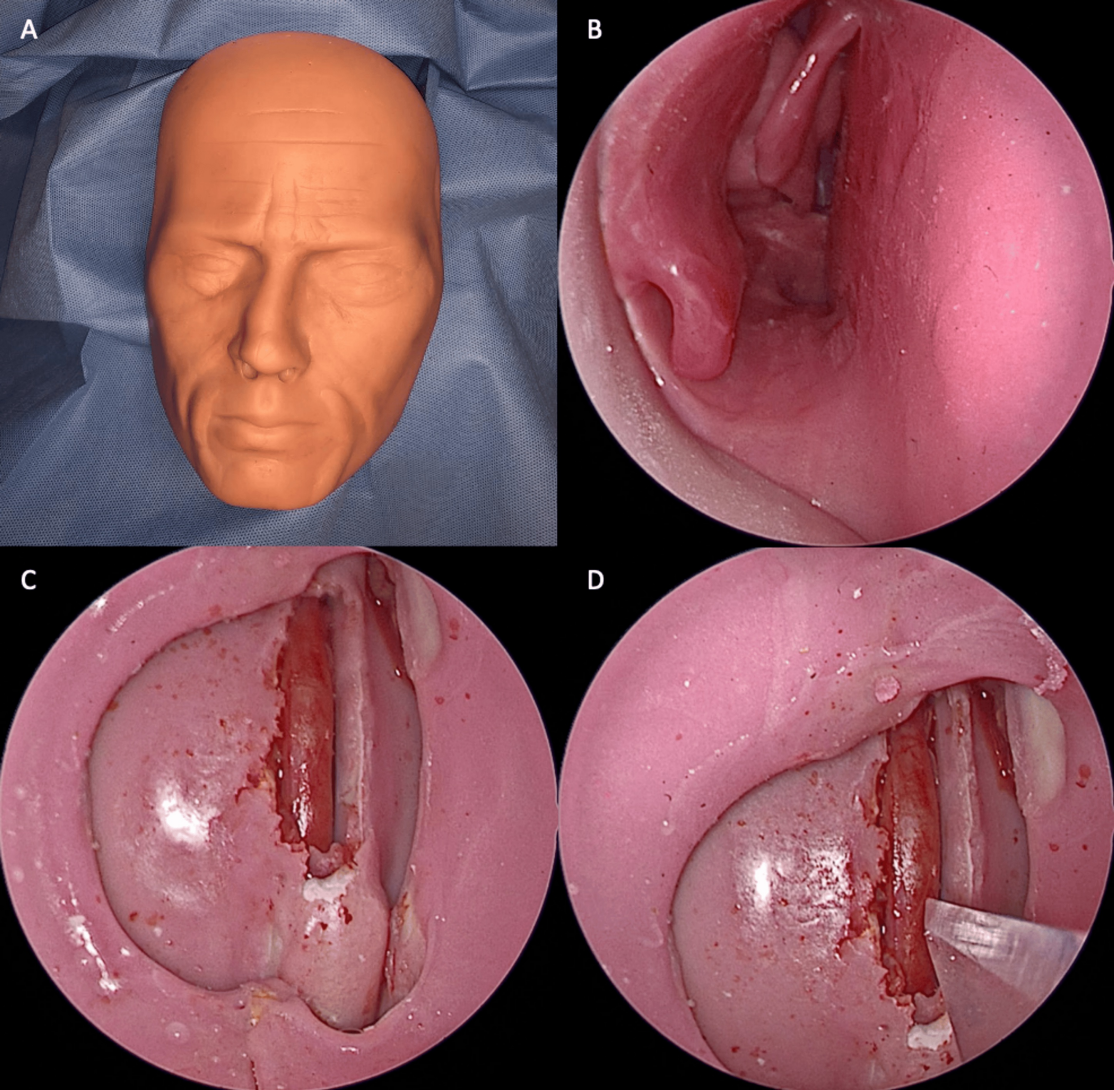
Model A – C: Anatomical model attached to the animal’s neck, on the common carotid artery. B: 0-degree scope view of the right nasal cavity of the anatomical model. C and D: Common carotid artery positioned on the sphenoid sinus, about to be injured with a no. 11 blade

A midline cervical incision of approximately 15 cm was performed to expose the common carotid artery. After careful dissection and isolation, the artery was surrounded by a hard plastic sheath, which facilitated its handling and stabilization. The vessel was then transposed into a three-dimensional printed sinonasal model (SIMONT, Pro Delphus, Pernambuco, Brazil), reproducing the parasellar and paraclival segments of the human ICA. Correct positioning of the artery was confirmed by endoscopic inspection using a 0-degree scope, ensuring that the surgical exposure reproduced the anatomical constraints of endonasal approaches (Figure 1).

In each artery, a standardized 3-4 mm vertical carotid injury was created with a No. 11 scalpel blade. Hemostasis was first attempted with a crushed autologous muscle patch (1×1 cm cervical muscle fragment, macerated between forceps and applied directly over the defect) (Video 1).

**Video 1:** Muscle patch ICA bleeding control with crushed muscle patch ICA: internal carotid artery

Subsequently, a T2 aneurysm clip (Mizuho®, Tokyo, Japan) was applied across the same lesion. Both maneuvers were performed sequentially in each injured artery. After bleeding control, vessel perfusion was evaluated using endonasal endoscopic intraoperative fluorescence videoangiography (Video 2).

**Video 2:** Artery patence post clipping 0-degree scope showing indocyanine green enhancing ICA blood flow after clipping the site of the arterial injury ICA: internal carotid artery

**Figure 2:**
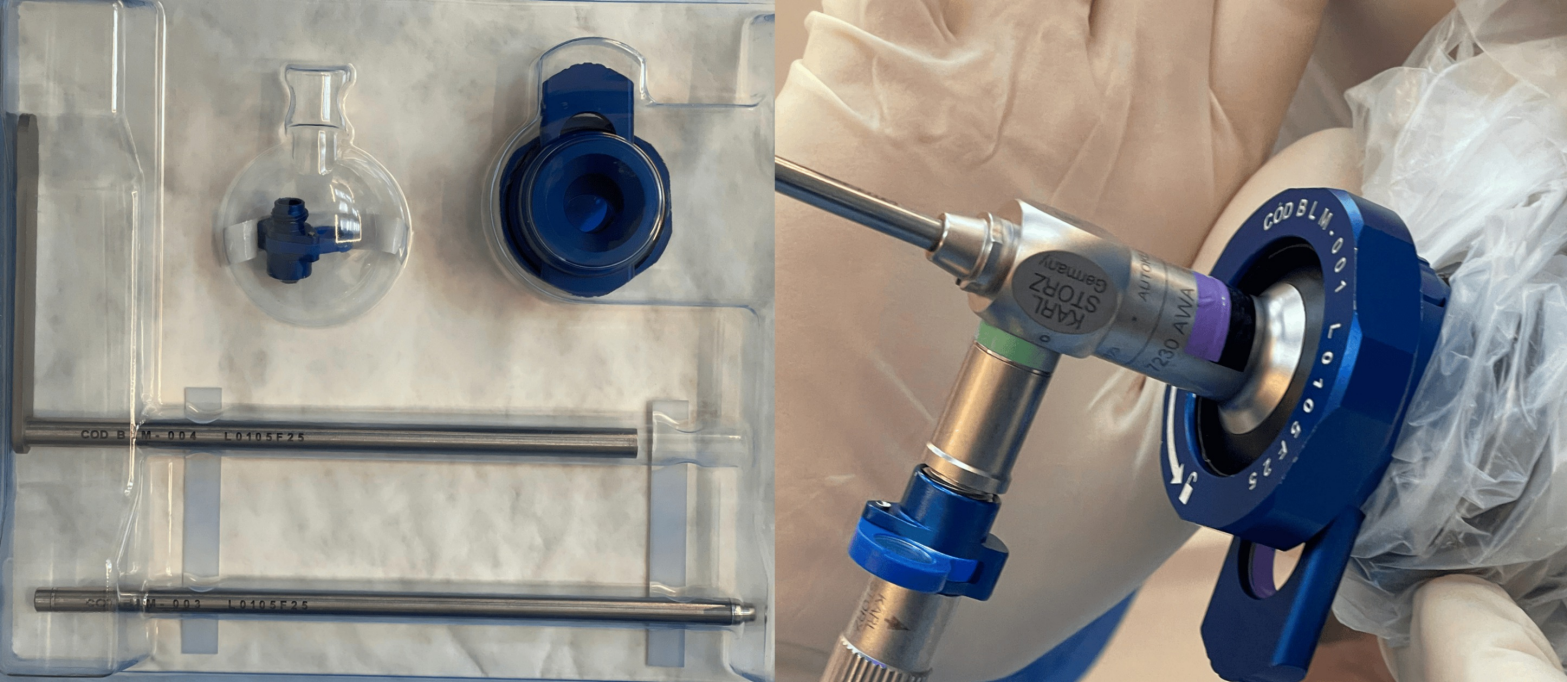
Endolumi kit Endolumi equipment attached to the scope and camera (Endolumi system, Practical Medical®, São Paulo, Brazil)

Either indocyanine green (5 mg IV; ICG/near-infrared (NIR) system, Karl Storz®, Tuttlingen, Germany) or fluorescein sodium (6 mg/kg IV; Endolumi system, Practical Medical®, São Paulo, Brazil) was administered, and distal flow was documented as patent or non-patent (Figure 2).

The choice of method depended on availability, and only one technique was used per artery.

Data collection

In the pilot phase, parameters collected included animal weight, MAP, and HR before and after injury, time from injury to cardiac arrest, infused fluid volume, bleeding volumes (after muscle and after clip), MAP before challenge, MAP before norepinephrine, MAP at arrest, and total blood loss until loss of oximetry signal. In the main experimental phase, outcomes included vessel patency after muscle and after clip application, as well as flow visualization with either ICG or FNa.

Statistical analysis

Data analysis was performed using SPSS Statistics version 27 (IBM Corp., Armonk, NY). For the pilot phase (n = 5), descriptive statistics were used, with continuous variables reported as mean, standard deviation (SD), median, and range. For the experimental phase, patency after muscle versus clip was compared using McNemar’s exact test for paired outcomes, and proportions were reported with 95% Wilson confidence intervals (CIs) [11]. Stratified analyses were also performed according to the fluorescence method (ICG vs. FNa). Due to unequal distribution between groups, ICG versus FNa performance was reported descriptively. A p-value <0.05 was considered statistically significant.

## Results

Pilot phase

Five pigs were studied in the pilot phase. Baseline MAP was 83.6 ± 4.2 mmHg, and HR was 91.8 ± 10.0 bpm. Following carotid injury, MAP decreased to 76.0 ± 8.0 mmHg, while HR increased slightly to 93.8 ± 7.5 bpm. The average time from injury to cardiac arrest was 38.7 ± 10.0 minutes. Time to the hemostatic challenge was 158 ± 133 seconds, with a pre-challenge MAP of 66.2 ± 1.8 mmHg. Norepinephrine was administered after 236 ± 128 seconds at a MAP of 61.6 ± 2.3 mmHg. At arrest, MAP dropped to 21.6 ± 5.4 mmHg. Mean infused volume was 1292 ± 149 ml, and total blood loss until loss of oximetry signal was 2700 ± 458 ml. Bleeding volumes were 232 ± 54 ml after muscle application and 258 ± 46 ml after clip placement. These findings confirmed that the porcine model consistently reproduced high-flow carotid bleeding with predictable hemodynamic deterioration.

Experimental phase

In the experimental phase (40 pigs, 80 carotid arteries), hemostasis was successfully achieved in all cases with both techniques. However, vessel patency differed significantly according to the method employed. After muscle patch application, patency was preserved in 79/80 arteries (98.8%, 95% CI: 93.3-99.8), whereas after clip placement it was preserved in 64/80 arteries (80.0%, 95% CI: 70.0-87.3). McNemar’s exact test confirmed that muscle repair maintained flow significantly more often than clip application (p<0.001).

Fluorescence angiography demonstrated that vascular patency was consistently preserved after muscle patch repair, while clip placement was associated with lower flow. Using ICG, patency was observed in 11 of 11 arteries (100%, 95% CI: 74.1-100) after muscle repair and in nine of 11 arteries (81.8%, 95% CI: 52.3-94.9) after clip application; this difference was not statistically significant (McNemar’s exact test, p = 0.50). With fluorescein, patency was documented in 68 of 69 arteries (98.6%, 95% CI: 92.2-99.7) following muscle repair compared with 55 of 69 arteries (79.7%, 95% CI: 68.8-87.5) after clip placement, a difference that reached statistical significance (McNemar’s exact test, p<0.001).

## Discussion

ICA injury during endoscopic endonasal skull base surgery remains one of the most feared intraoperative complications. Although its incidence is low - reported to be between 0.3% and 0.9% in large series - the consequences can be devastating, including exsanguination, stroke, pseudoaneurysm formation, and death. Traditional training methods, based on sporadic real-life exposure, are inadequate to prepare surgeons for such catastrophic events. This has driven the development of simulation-based platforms to provide reproducible and safe training opportunities [1].

The first high-flow ICA injury simulation model was introduced by Valentine and Wormald using live sheep, demonstrating the feasibility of creating reproducible arterial bleeding in a narrow endonasal corridor [3]. Perfusion-based cadaveric models have since been developed, providing accurate anatomy but lacking the dynamic physiology and hemodynamics of live animals [1]. Synthetic laser-sintered skull base models have also been described. While they provide accurate anatomical reproduction, they lack a true arterial wall and therefore cannot reproduce the biological interactions that occur during hemostasis [1]. In addition, they are unable to replicate dynamic physiological responses such as arterial pressure, coagulation cascades, and the pressurized bleeding of a living system.

Our findings expand on this evidence by validating a live porcine model, which offers additional advantages in availability in Latin America and vascular dimensions that approximate the parasellar and paraclival ICA. Importantly, the porcine common carotid artery has a diameter similar to the human ICA at the skull base, supporting its translational fidelity. Consistent with prior reports, both crushed skeletal muscle and aneurysm clips achieved immediate hemostasis [3]. However, our results demonstrate that muscle repair preserved luminal patency in 98.8% of cases, compared with 80% after clip placement. This aligns with the work of Rajiv et al., who showed that muscle extract promotes platelet aggregation and clot stability, supporting its biological role in vascular repair [5]. Conversely, clip application, while effective for bleeding control, risks mechanical stenosis or occlusion, as reported in both experimental and clinical settings [2]. These findings reinforce current expert recommendations that muscle patches should be prioritized when feasible, reserving clips for select cases [1].

A key advantage of live-animal simulation compared with cadaveric and synthetic models is the biological interaction between vascular structures and hemostatic materials. Only in living tissue can platelet aggregation and thrombus formation occur under physiological conditions, modulated by endothelial integrity, coagulation cascades, and arterial pressure. In this study, the macerated muscle patch served not only as a mechanical tamponade but also as a biologically active substrate, facilitating platelet adhesion and interaction with tissue factors released at the arterial wall [5]. Such processes cannot be replicated in perfusion-based cadaveric models, where coagulation pathways are absent, or in synthetic models, which lack endothelial and muscular substrates [1]. The live porcine model, therefore, combines anatomical realism, hemodynamic fidelity, and biological responses, establishing it as a high-fidelity platform for testing hemostatic strategies and for training surgeons in scenarios that closely reproduce intraoperative physiology [3].

Another innovation in this study was the integration of intraoperative fluorescence videoangiography. ICG and FNa are well established in cerebrovascular neurosurgery for assessing flow after aneurysm clipping [6,7,12,13], and recent reports have extended their use to endoscopic skull base surgery [7]. In our series, both modalities were able to confirm vessel patency after hemostasis. Although the number of arteries assessed with FNa was substantially higher than those assessed with ICG, statistical analysis showed that Endolumi FNa demonstrated adequate performance and did not show a reduction in visualization quality when compared with the widely used NIR/ICG system. Moreover, the fluorescein-based equipment represents a more economically accessible technology, which may facilitate broader implementation in training and clinical practice, particularly in resource-limited settings.

Educational implications

Simulation models not only validate hemostatic techniques but also provide critical educational value [14]. Donoho et al. showed that cadaveric perfusion-based simulation significantly improved surgeon performance, reducing blood loss and time to hemostasis, and enhancing confidence during ICA injury management [15]. Similarly, Maza et al. demonstrated that a simplified 3D laser-sintered model improved psychomotor skills, shortened time to hemostasis, and increased trainee confidence [16]. These findings highlight how structured simulation can accelerate acquisition of crisis-management skills for rare, high-risk complications.

Our results extend these observations by demonstrating that a live porcine model not only reproduces the hemodynamic reality of ICA injury but also discriminates between repair strategies - specifically, crushed muscle versus aneurysm clip. This capacity for comparative validation supports its utility both as a technical benchmarking platform and as an educational intervention to prepare surgeons for catastrophic intraoperative bleeding scenarios. When considered alongside existing cadaveric and synthetic models, the porcine live-tissue platform offers advantages by integrating anatomic fidelity, hemodynamic realism, and biologic responses such as platelet aggregation. This strengthens its role as a valuable complement to other training tools within comprehensive skull base surgery curricula.

Limitations and future directions

As with other live-animal simulation models, this approach is limited by cost, ethical concerns, and anatomical differences between swine and humans. Nonetheless, no synthetic or cadaveric model has yet reproduced the dynamic physiology, blood pressure, and catastrophic nature of ICA bleeding. Future directions include multicenter validation, integration of team-based crisis simulations, and comparative studies with emerging augmented reality and 3D-printed platforms.

## Conclusions

This study describes the development and validation of a live porcine model for endoscopic endonasal management of catastrophic ICA injury. The model proved to be reproducible, anatomically and physiologically realistic, and capable of distinguishing between different hemostatic strategies. Crushed skeletal muscle consistently preserved vessel patency at significantly higher rates than aneurysm clip application, supporting its use as an effective hemostatic method within this model. Beyond the comparison of techniques, the porcine live model demonstrated biological responses not present in cadaveric or synthetic platforms. The interaction between macerated muscle and the arterial wall promoted platelet aggregation and thrombus formation under true physiological conditions - an effect that cannot be reproduced in inanimate models. This highlights the superiority of live-animal simulation in replicating the complexity of vascular repair and in providing a high-fidelity environment for testing and training.
